# Involvement of the P2X7 receptor in the migration and metastasis of tamoxifen-resistant breast cancer: effects on small extracellular vesicles production

**DOI:** 10.1038/s41598-019-47734-z

**Published:** 2019-08-12

**Authors:** Miso Park, Jieun Kim, Nguyen T. T. Phuong, Jung Gyu Park, Jin-Hee Park, Yong-Chul Kim, Moon Chang Baek, Sung Chul Lim, Keon Wook Kang

**Affiliations:** 1 College of Pharmacy and Research Institute of Pharmaceutical Sciences, Seoul National University, Seoul, 08826 Republic of Korea; 2 School of Life Sciences, Gwangju Institute of Science & Technology, Gwangju, 61005 Republic of Korea; 3 Department of Biochemistry, School of Medicine, Kyungpook National University, Daegu, 41944 Republic of Korea; 4 Department of Pathology, College of Medicine, Chosun University, Gwangju, 61452 Republic of Korea

**Keywords:** Cell invasion, Breast cancer

## Abstract

Tamoxifen (TAM) is the standard anti-hormonal therapy for estrogen receptor-positive breast cancer. However, long-term TAM therapy can make acquisition of TAM resistance and there are still no solutions to treat TAM-resistant breast cancer. In this study, we found that protein and mRNA expression of the P2X purinoreceptor 7 (P2X7) was higher in tamoxifen resistant MCF-7 (TAMR-MCF-7) cells than in control MCF-7 cells. P2X7 inhibition potently inhibited the migration of TAMR-MCF-7 cells and the liver metastasis burden of TAMR-MCF-7 cells in the spleen-liver metastasis experiment. However, the P2X7 antagonist did not affect protein expression of matrix metalloproteinase (MMP)-2, MMP-9, and epithelial-mesenchymal transition markers. Here our data indicate a link between small extracellular vesicles (sEV) and P2X7, and suggest a new mechanism of metastasis in TAM-resistant breast cancer cells through P2X7 receptors. The migration of TAMR-MCF-7 cells was increased in a concentration-dependent manner by purified sEV treatment. The number of secreted sEVs and the protein levels of CD63 in TAMR-MCF-7 cells were decreased by the P2X7 antagonist, showing that P2X7 influences the production of sEV. Our results suggest that inhibiting the P2X7 could be considered for metastasis prevention in TAM-resistant cancer patients.

## Introduction

Breast cancer is the most common malignancy in women in the developed countries and estrogen is a key hormone causing uncontrolled growth of estrogen receptor (ER)-positive and luminal type breast cancer^1^. Anti-hormone therapy can be used as first-line chemotherapy for ER-positive breast cancer. Tamoxifen (TAM), a non-steroidal anti-estrogen, is the most widely prescribed agent for ER-positive breast cancer patients. Despite an initial response, most patients develop resistance to TAM, and relapsed tumor growth may occur^2^. In addition, TAM-resistant cancer cells show increased migration and invasiveness, and eventually have increased metastatic potential^3,4^. The 5-year survival rate for breast cancer was 89.7% in 2008–2014, which is relatively higher than for other cancers, but the 5-year survival rate after metastasis drops to 27.0%^5^. However, the proper treatment option for such cases has not yet been established.

Recently, the tumor microenvironment has been attracting attention as an important factor in cancer progression and anticancer drug resistance^6^. Owing to repeated growth and death processes of cancer cells, purine nucleotides such as Adenosine triphosphate (ATP) are abundant around tumor tissues^7^. High levels of extracellular purine nucleotides stimulate unique signaling through the purinergic receptor, which plays an important role in the interaction between host and cancer^8^. Purinergic receptors consist of P1 and P2 receptors, and P2 receptors are further divided into G-protein coupled P2Y receptors and ligand-gated ion channel P2X receptors^9^. The P2X7 receptor is a cationic ion channel receptor activated by ATP, and is distributed in macrophages, microglial cells, and pancreas, skin, bone, and kidney cells^10^. Moreover, the receptor is frequently overexpressed in various types of tumor tissues including breast, thyroid, and prostate cancer^11^. The most important role of P2X7 is ATP-dependent activation of the inflammasome in immune cells. The activated P2X7 bound to ATP triggers either excretion of intracellular potassium or uptake of extracellular calcium, which is responsible for the subsequent activation of the NLR family, pyrin domain containing 3 (NLRP3) inflammasome components consisting of NLRP3, apoptosis-associated speck-like protein containing a caspase-recruitment domain (ASC), and pro-caspase-1^12^. It has also been reported that P2X7 activity is involved in the proliferation, cell death and metastasis of cancer cells^13^. However, the role of P2X7 in TAM-resistant breast cancer has not been studied.

Extracellular vesicles (EV) show spherical biomembrane structures and are divided into microvesicles (or microparticles) and exosomes^14^. The main differences between exosomes and microparticles are size and biosynthesis process. Microvesicles, 50–3,000 nm in size, are derived from membrane-shedding extracellular vesicles, whereas 30–100-nm exosomes are derived from multivesicular body-derived extracellular vesicles^14^. 30–200 nm small extracellular vesicles (sEV) including exosomes are widely distributed in body fluids such as blood, urine, ascites, and amniotic fluid, and they contain diverse bioactive cellular components including mRNAs, miRNAs, DNA fragments, proteins, and lipids^15^. In particular, sEVs secreted from cancer cells can transport bioactive cellular components into recipient cells and play an important role in the regulation of diverse cell signaling and thereby affect progression, angiogenesis, metastasis, and immune response avoidance of cancer^16–18^. In the present study, we found for the first time that P2X7 was overexpressed in TAM-resistant breast cancer cells and investigated its functional roles using a P2X7 selective antagonist. We further sought to clarify the mechanistic basis for the anti-migration effect of the P2X7 antagonist, focusing on sEV secretion.

## Results

### Enhanced expression of P2X7 in TAMR-MCF-7 cells

**Figure 1:**
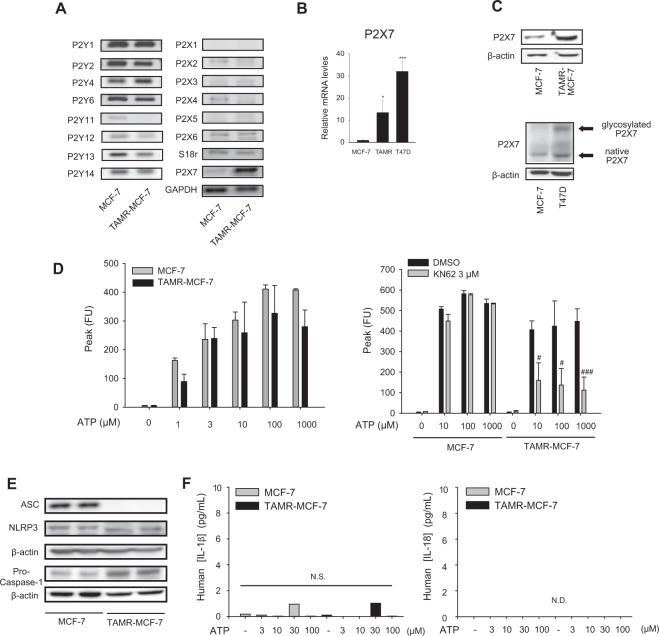
Enhanced expression of P2X7 Receptor in TAMR-MCF-7 cells. (**A**) mRNA Expression of P2Y receptors and P2X receptors in MCF-7 and TAMR-MCF-7 cells. mRNA levels were determined by RT-PCR analyses. (**B**) Quantitative mRNA expression of P2X7 receptors in MCF-7, TAMR-MCF-7, and T47D cells (n = 11). (**C**) Protein Expression of P2X7 in MCF-7, TAMR-MCF-7, and T47D cells (n = 3). (**D**) ATP-induced P2X7 activation in MCF-7 and TAMR-MCF-7 cells. [Ca^2+^]i were measured after treating both the cell types with ATP (1–1000 μM) in ATP-free buffer condition (Left). MCF-7 and TAMR-MCF-7 cells were pretreated with 3 μM KN62, a selective P2X7 antagonist, and then incubated with ATP (10, 100 and 1000 μM) (Right)(n = 4). (**E**) Protein expression of inflammasome units (ASC, NLRP3 and pro-caspase-1) in MCF-7 and TAMR-MCF-7 cells (n = 4). (**F**) IL-1β and IL-18 production in culture media by detected ELISA analyses (N.S., not significant. N.D., not detected). All data represent the mean ± SE (*p < 0.05, ***p < 0.005, significant as compared to MCF-7 cells; ^#^ < 0.05, ^###^ < 0.005, significant as compared to TAMR-MCF-7 control group).

Purinergic receptors are activated by extracellular purine nucleotides such as ADP or ATP^9^. When we determined the mRNA levels of diverse purinergic receptors by RT-PCR analyses, the basal mRNA expression of P2X7 was selectively upregulated in TAMR-MCF-7 cells compared to parental MCF-7 cells (Fig. 1A). Although both T47D and MCF-7 breast cancer cell lines are considered ER-positive, T47D cells are a relatively more TAM-resistant clone^19,20^. As shown in Fig. 1B, the P2X7 mRNA level was significantly increased in TAMR-MCF-7 cells and T47D cells compared to MCF-7 cells (13.5 and 32.1 fold, respectively). Immunoblot assay also confirmed the result (Fig. 1C). In addition, the glycosylated form of P2X7 was also observed in T47D cells (Fig. 1C).

We then compared P2X7 receptor activity between MCF-7 and TAMR-MCF7-cells. Because P2X7 activation induces an intracellular calcium increase, we first determined the relative increase in calcium using a FLIPR Ca²^+^ assay kit. In an ATP-free buffer condition, the basal calcium florescence in TAMR-MCF-7 cells was not distinct from that in MCF-7 cells. Treatment of MCF-7 and TAMR-MCF-7 cells with 3–1,000 μM ATP increased intracellular calcium ([Ca^2+^]i) in a concentration-dependent manner, and the maximal increase was found at 100 μM in both cell types (Fig. 1D). Interestingly, ATP-induced [Ca^2+^]i increases were potently suppressed by KN62, a selective P2X7 antagonist^21^, in TAMR-MCF-7 cells, but not in MCF-7 cells. The results demonstrate that P2X7 plays a major role in ATP-dependent calcium signaling only in TAMR-MCF-7 cells.

ATP-dependent inflammasome activation (NLRP3 inflammasome) in immune cells is representative pharmacodynamic effect of P2X7^12^. When we assessed the protein expression of NLRP3 inflammasome components in MCF-7 and TAMR-MCF-7 cells, ASC, one of the key components of inflammasome, was deficient in TAMR-MCF-7 cells (Fig. 1E). Moreover, secretion of Interleukin-1β (IL-1β) and Interleukin-18 (IL-18), the end products of inflammasome activation, was not observed in either MCF-7 or TAMR-MCF-7 cells (Fig. 1F). These data imply that P2X7 is responsible for ATP-mediated cation flux but not inflammasome activation in TAMR-MCF-7 cells.

### Role of P2X7 in proliferation of MCF-7 and TAMR-MCF-7 cells

**Figure 2:**
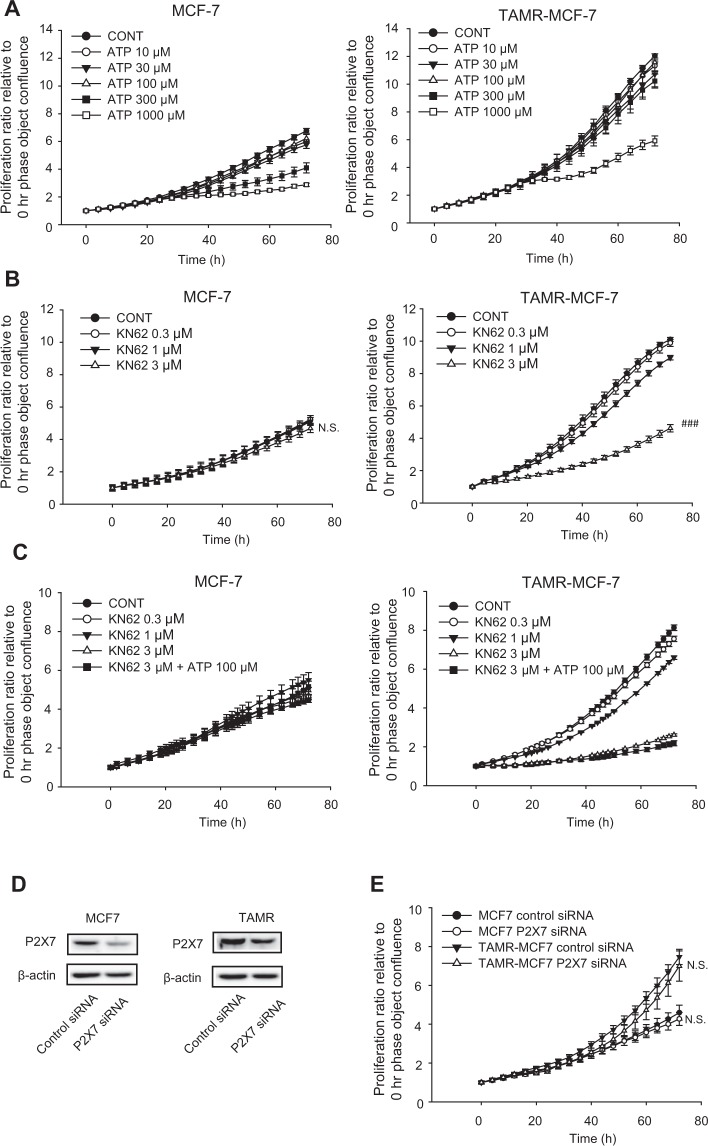
Limited role of P2X7 in cell proliferation of MCF-7 and TAMR-MCF-7 cells. (**A**) Effects of ATP on cell proliferation of MCF-7 and TAMR-MCF-7 cells (n = 5). (**B**) Effects of KN62 (0.3–3 μM) on cell proliferation of MCF-7 and TAMR-MCF-7 cells (n = 6). (**C**) Effects of KN62 (0.3–3 μM) cotreatment on cell proliferation of MCF-7 and TAMR-MCF-7 cells incubated with 100 μM ATP (n = 6). (**D**) Inhibition of P2X7 expression by P2X7 siRNA treatment. (**E**) Effects of P2X7 silencing on cell proliferation of MCF-7 and TAMR-MCF-7 cells (n = 10). All data represent the mean ± SE (^###^p < 0.005, significant as compared to TAMR-MCF-7 control group).

It has been reported that P2X7 expressed in cancer cells is involved in diverse phenotypes of cancer including proliferation, migration and invasion^22,23^. We previously reported that epithelial-mesenchymal transition (EMT)-associated cell migration is enhanced in TAMR-MCF-7 cells compared to control MCF-7 cells^24^. When we assessed relative cell proliferation, the proliferation rate of TAMR-MCF-7 cells was faster than that of MCF-7 cells, and the proliferation rate of MCF-7 and TAMR-MCF-7 cells was not affected by ATP treatment (up to 100 μM) (Fig. 2A). However, cell proliferation was partially but significantly suppressed by 3 μM KN62, a P2X7 antagonist, in TAMR-MCF-7 cells but not in MCF-7 cells (Fig. 2B). Because KN62 is a competitive antagonist of P2X7, we further assessed the combined effect of KN62 with 100 μM ATP. As shown in Fig. 2C, ATP did not reverse the KN62-mediated inhibitory effect on cell proliferation in TAMR-MCF-7 cells, suggesting that the anti-proliferative effect of KN62 is not due to the P2X receptor.

Therefore, we next used the P2X7 receptor siRNA system to clarify the relationship between the P2X7 receptor and TAMR-MCF-7 cell proliferation. Although the expression of the P2X7 receptor was reduced by transfection with P2X7 siRNA compared to control siRNA (Fig. 2D), P2X7 silencing did not affect the proliferation of MCF-7 and TAMR-MCF7 cells (Fig. 2E). These results indicate that the proliferation of TAMR-MCF-7 cells may not depend on P2X7 activity.

### Involvement of P2X7 in migration of TAMR-MCF-7 cell

**Figure 3:**
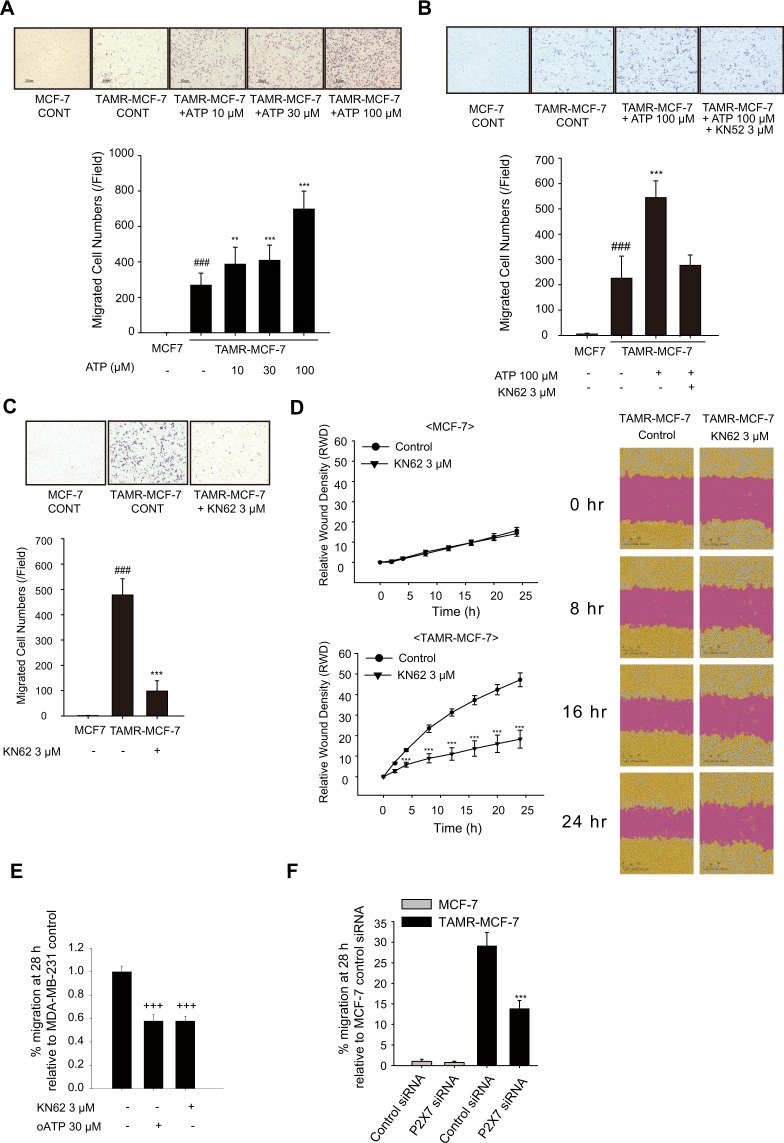
Involvement of P2X7 in migration of TAMR-MCF-7 cells. (**A**) Effects of ATP on cell migration of MCF-7 and TAMR-MCF-7 cells. Both the cell types were treated with ATP (10–100 μM) for 18 h. Cell migration was evaluated by transwell migration assays (n = 8). (**B**) Effect of KN62 on ATP-induced cell migration of TAMR-MCF-7 cells. MCF-7 and TAMR-MCF-7 cells cultured on transwell were treated with 100 μM ATP in the presence or absence of 3 μM KN62 for 18 h (n = 8). (**C**) Effect of KN62 (3 μM) on the basal cell migration of TAMR-MCF-7 cells (n = 8). (**D**) Wound healing assay. Cell migration was confirmed by wound healing assay (n = 4). Images were taken by Incucyte Zoom. (**E**) Effect of oATP (30 μM) and KN62 (3 μM) on the basal cell migration of MDA-MB-231 cells (n = 4). (**F**) Effects of P2X7 silencing on cell migration of MCF-7 and TAMR-MCF-7 cells (n = 4). All data represent the mean ± SE (^###^p < 0.005, significant as compared to control MCF-7 cells; **p < 0.01, ***p < 0.005, significant as compared to TAMR-MCF-7 control cells; ^+++^p < 0.005, significant as compared to MDA-MB-231 control cells).

We then investigated the role of P2X7 in the migration of TAMR-MCF-7 cells. In comparison to MCF-7 cells, cell migration was highly elevated in TAMR-MCF-7 cells in the transwell migration assay (Fig. 3A). To determine whether the enhanced migration capability of TAMR-MCF-7 cells is due to P2X7 activation, the migration ability of TAMR-MCF-7 cells was assessed in the presence of ATP and/or KN62. The migration of TAMR-MCF-7 cells was increased in a concentration-dependent manner by treatment with 10–100 μM ATP; in particular, 100 μM ATP enhanced migration by more than 2-fold (Fig. 3A). In addition, ATP-driven cell migration in TAMR-MCF-7 cells was abolished by the P2X7 antagonist KN62 (Fig. 3B). Also, transwell migration and wound healing assays revealed that the basal migration of TAMR-MCF-7 cells was suppressed by KN62 treatment (Fig. 3C,D). The anti-migratory effect of KN62 was confirmed by another selective P2X7 inhibitor, Adenosine 5′-triphosphate, periodate oxidized sodium salt (oATP)(Fig. S1A). To clarify the migration ability promoted by P2X7R, we silenced the P2X7 receptor using specific siRNA in TAMR-MCF-7 cells (Fig. 3F and transwell migration images were shown in Fig. S1D). As expected, the migration ability of TAMR-MCF-7 cells was significantly decreased by silencing P2X7. The data suggest that P2X7 activation in TAMR-MCF-7 cells is critical in the enhanced migration of TAMR-MCF-7 cells.

We further tested if P2X7 is related with cell migration of triple-negative breast cancer (TNBC) cell line, MDA-MB-231 cells. Even though the expression of P2X7 in MDA-MB-231 is not relatively higher than MCF-7 cells (Fig. S1C), the migration of MDA-MB-231 cells was also suppressed by KN62 or oATP treatment (Fig. 3E and transwell migration images were shown in Fig. S1B), indicating that P2X7 in MDA-MB-231 is also related to cell migration of MDA-MB-231 cells.

### Effect of P2X7 antagonist on spleen-liver metastasis of TAMR-MCF-7 cells

**Figure 4:**
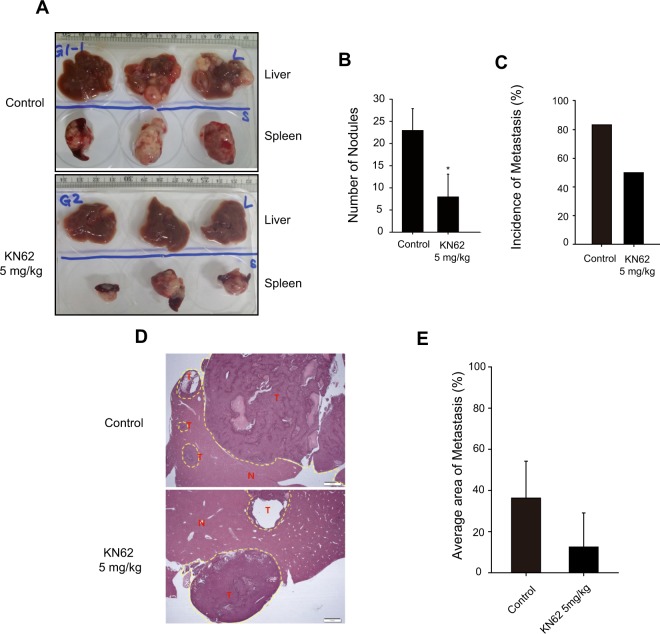
Effect of P2X7 antagonist on metastatic tumor burden. (**A**) Representative tumor burden images of livers (upper) and spleens (lower). (**B**) Number of metastatic liver nodules per mouse (n = 6). (**C**) Incidence of liver metastasis in control and KN62-treated group (n = 6). (**D**) Representative H&E staining images of liver sections from each group. (**E**) Average tumor tissue area in liver sections (n = 6). All data represent the mean ± SE (*p < 0.05, significant compared as control group).

Because P2X7 is actively involved in cell migration of TAMR-MCF-7 cells *in vitro*, we then estimated the effect of KN62 in the mouse spleen-liver metastasis model. We have previously shown that mice implanted with TAMR-MCF-7 cells in the spleen showed a remarkable liver metastatic tumor burden^24^. Balb/c-nu mice were divided into two groups, a spleen implantation of TAMR-MCF-7 cells and vehicle-injected group (group 1), and a spleen implantation of TAMR-MCF-7 cells and 5 mg/kg KN62-injected group (group 2). The mice implanted with TAMR-MCF-7 cells showed aggressive tumors in the spleen and a high frequency (88.9% incidence) of macroscopic metastases in the liver (Fig. 4A). Intraperitoneal injection of KN62 (5 mg/kg/day) significantly reduced the liver metastatic tumor burden (number of nodules, Fig. 4B), and only three of six mice displayed metastases (Fig. 4C). When we compared the average area percentage of metastasis in metastasis-positive samples, the tumor area was diminished by KN62 injection (Fig. 4D,E). These results suggest that P2X7 could be a therapeutic target for tumor metastasis in TAM-resistant breast cancer.

### P2X7-mediated cell migration is not dependent on EMT or expression of MMP-2/9

**Figure 5:**
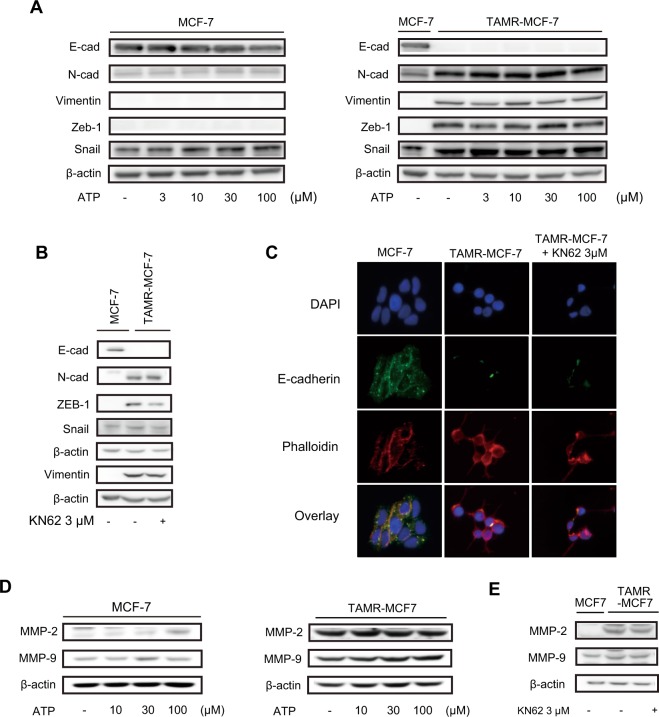
No involvement of EMT or MMP-2/9 expression in P2X7-mediated cell migration. (**A**) Expression of EMT markers (E-cadherin, N-cadherin, Vimentin, Zeb-1, and Snail) in MCF-7 and TAMR-MCF-7 cells after treatment with 3–100 μM ATP for 48 h. (**B**) Expression of EMT markers (E-cadherin, N-cadherin, Vimentin, Zeb-1, and Snail) in MCF-7 and TAMR-MCF-7 cells after treatment with 3 μM KN62 for 48 h. (**C**) Immunocytochemical stainings of DAPI, E-cadherin and phalloidin in MCF-7 and TAMR-MCF-7 cells (x 20). (**D**) Expression of MMP-2 and MMP-9 in MCF-7 and TAMR-MCF-7 cells after treatment with 10–100 μM ATP for 48 h. (**E**) Expression of MMP-2 and MMP-9 in MCF-7 and TAMR-MCF-7 cells after treatment with 3 μM KN62 for 48 h.

Because the function of P2X7-mediated cell migration was confirmed *in vitro* and *in vivo*, we further sought to clarify downstream events of P2X7 in TAMR-MCF-7 cells. EMT is a process in which an epithelial cell acquires the characteristics of a mesenchymal cell. The cell switches from a polarized epithelial phenotype to a highly motile mesenchymal phenotype; this change is important for cell migration and metastasis of cancer cells^25^. Based on a previous report showing that P2X7 stimulates cell migration and invasion via the activation of EMT-related genes in prostate cancer cells^11^, EMT markers in MCF-7 and TAMR-MCF-7 cells were estimated. We have previously reported that TAMR-MCF-7 cells show typical EMT features^24,25^. Immunoblot analyses confirmed the loss of E-cadherin (a representative epithelial adhesion marker) and upregulation of N-cadherin, vimentin, Zeb-1, and Snail (mesenchymal marker proteins) in TAMR-MCF-7 cells (Fig. 5A, right). Incubation of MCF-7 or TAMR-MCF-7 cells with ATP (3–100 μM) for 48 h did not cause any significant changes in protein expression of EMT markers (Fig. 5A). Moreover, exposure of TAMR-MCF-7 cells to the P2X7 antagonist KN62 for 48 h did not affect the basal expression of EMT markers except Zeb-1 levels in TAMR-MCF-7 cells (Fig. 5B). Immunocytochemistry confirmed that the reduced E-cadherin immunofluorescence in TAMR-MCF-7 cells did not recover by 48 h after KN62 exposure (Fig. 5C). We further determined whether KN62 or oATP affect the expression levels of EMT markers in MDA-MB-231 cells. P2X7 inhibition did not change the expression of EMT markers in MDA-MB-231 cells (Fig. S1E).

Matrix metalloproteinase (MMP)-2 and MMP-9 are essential for the degradation of cell surface proteins and help migratory cells to invade the basement membrane^26^. The protein levels of MMP-2 and MMP-9 were not significantly changed by ATP or KN62 (Fig. 5D,E). Hence, P2X7-mediated migration and the metastatic potential of TAMR-MCF-7 cells do not seem to be related to EMT or MMP signaling.

### Role of P2X7 in sEV secretion in TAMR-MCF-7 cells

It has been reported that enhanced intracellular calcium as a key secondary signal transducer promotes sEV secretion^27^. P2X7 activation stimulates calcium influx and subsequently results in the secretion of sEVs in macrophages and dendritic and neuroblastoma cells^27^. Because ATP-dependent calcium influx was potently inhibited by a P2X7 antagonist in TAMR-MCF7 cells but not in MCF-7 cells (Fig. 1C), we further investigated the effects of KN62 on sEV secretion in TAMR-MCF-7 cells. The secreted sEVs in both MCF-7 and TAMR-MCF-7 cells were isolated from culture media, and the size and number of sEVs were characterized (Fig. S2A). Although the average size of sEVs was slightly larger in TAMR-MCF-7 cells, there was no significant difference in the number of sEVs between MCF-7 and TAMR-MCF-7 cells (Figs S2A and 6A).

**Figure 6:**
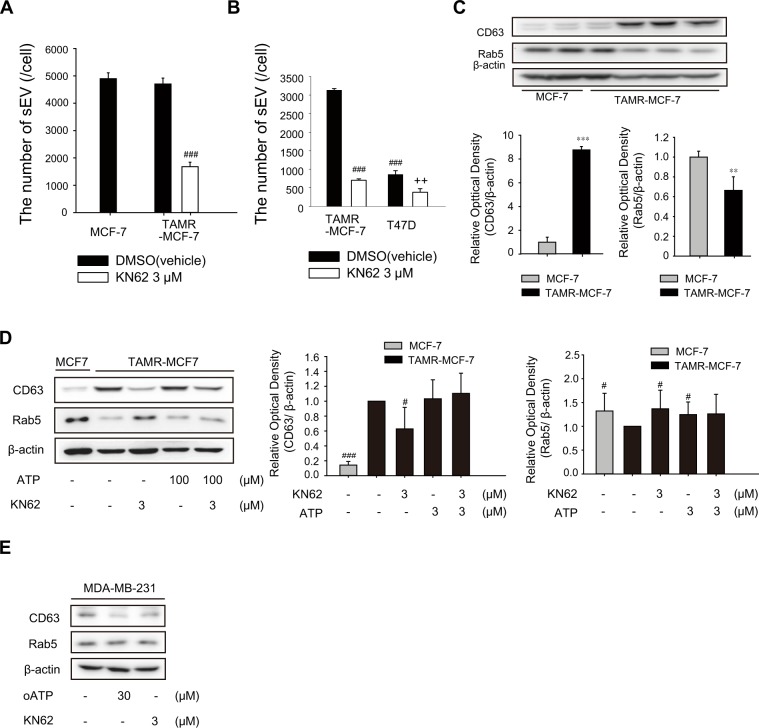
Role of P2X7 in sEV secretion in TAMR-MCF-7 cells. (**A**) Effect of KN62 on the production of sEVs in MCF-7 cells and TAMR-MCF-7 cells. The number sEVs was determined by nanoparticle tracking analysis after treatment of cells with 3 μM KN62 for 24 h (normalized by initial number of plating cells)(n = 3). (**B**) Effect of KN62 on the production of sEVs in T47D and TAMR-MCF-7 cells (n = 3). (**C**) Protein expression of CD63 and Rab5 in MCF-7 and TAMR-MCF-7 cells (n = 3). (**D**) Effects of KN62 on the protein expression of CD63 (n = 3) and Rab5 (n = 5) in MCF-7 and TAMR-MCF-7 cells. (**E**) Effects of KN62 on the protein expression of CD63 (n = 6) and Rab5 (n = 6) in MDA-MB-231 cells. (**p < 0.01, ***p < 0.005, significant as compared to MCF-7 cells; ^#^p < 0.05, ^###^p < 0.005, significant as compared to TAMR-MCF-7 control group; ^++^p < 0.01, significant as compared to T47D control group).

Interestingly, sEV secretion was suppressed by KN62 treatment in TAMR-MCF-7 cells (Fig. 6A). As depicted in Fig. 1C, T47D cells showed higher P2X7 expression relative to MCF-7 cells, and it has been reported that migration of T47D cells is mediated by P2X7^13^. When we performed sEV counting in both TAMR-MCF-7 cells and T47D cells, the number of secreted sEV was decreased in T47D cells compared to TAMR-MCF-7 cells (Fig. 6B); however, the number of secreted sEV was significantly reduced by KN62 treatment in both the cell types.

We then quantified the representative sEV-specific markers required for secretion and production of sEVs. The protein expression level of rab5 required for the production of sEVs was higher in MCF-7 cells, but the expression level of CD63 mediating the secretion of sEVs was higher in TAMR-MCF-7 cells than in MCF-7 cells (Fig. 6C). Intriguingly, the elevated expression of CD63 in TAMR-MCF-7 cells was almost completely decreased by KN62; in contrast, Rab5 expression was restored by the P2X7 antagonist (Fig. 6D). Another selective P2X7 inhibitor, oATP has also similar effects (Fig. S2B). In MDA-MB-231 cells, a TNBC cell line, the protein level of CD63 was also diminished by oATP or KN62 treatment (Fig. 6E). These results suggest that P2X7 is involved in the secretion of sEVs, presumably through changes in CD63 and Rab5 expression in invasive breast cancer cells.

### Role of sEV in cell migration of TAMR-MCF-7 cells

**Figure 7:**
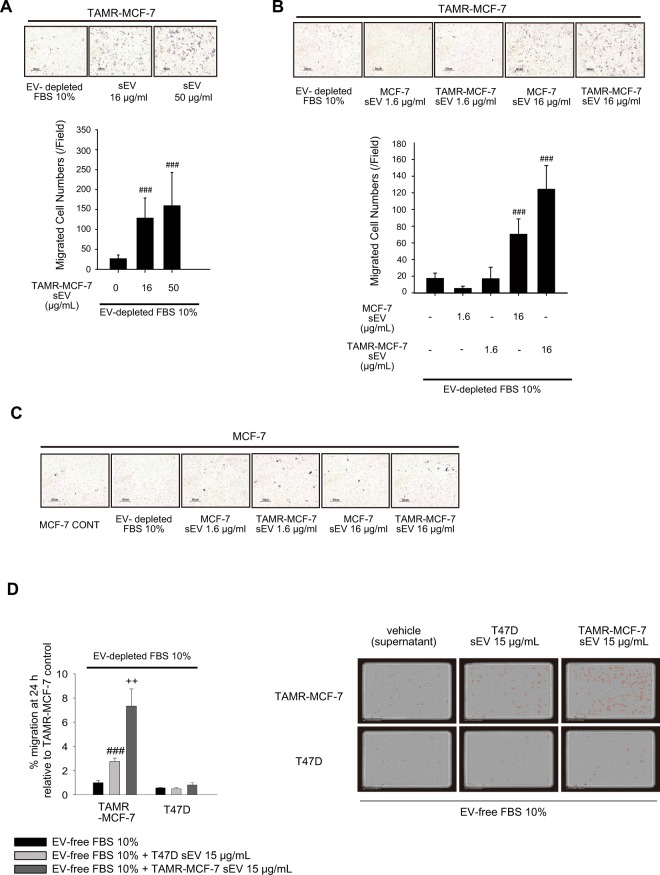
Essential role of sEV in cell migration of TAMR-MCF-7 cells. (**A**) Migration of TAMR-MCF-7 cells after incubation with TAMR-MCF-7 sEV (16 and 50 μg/mL) for 18 h (n = 10). (**B**) Migration of TAMR-MCF-7 cells after incubation with MCF-7 or TAMR-MCF-7 sEV (1.6 and 16 μg/mL) for 18 (n = 10). (**C**) Migration of MCF-7 cells after incubation with MCF-7 or TAMR-MCF-7 sEV (1.6 and 16 μg/mL) for 18 h (n = 10). Cell migration was evaluated by Transwell migration assay in sEV-depleted FBS condition. (**D**) Migration of T47D and TAMR-MCF-7 cells after incubation with T47D and TAMR-MCF-7 sEV (15 μg/mL) for 24 h (n = 3). Migrated cells were marked in red circle by image analysis. Images were taken by Incucyte Zoom. All data represent the mean ± SE (^###^p < 0.005, significant as compared to TAMR-MCF-7 cells in sEV depleted condition, ^++^p < 0.01, significant as compared to TAMR-MCF-7 cells in T47D sEV 15 μg/mL condition).

sEVs play a critical role in cancer cell migration and metastasis, either by transferring tumor promoting proteins and nucleotides or by forming a premetastatic niche^28,29^. Hence, we evaluated the effects of sEVs in the migration of MCF-7 and TAMR-MCF-7 cells. To exclude the biased effect of fetal bovine serum (FBS)-derived EV, MCF-7 and TAMR-MCF-7 cells were cultured in an EV-free FBS condition. When TAMR-MCF-7 cells were exposed to isolated sEVs from TAMR-MCF-7 cells, the basal cell migration increased in a concentration-dependent manner, and an approximately 4.9- and 6.1-fold increase was seen with 16 and 50 μg/mL TAMR-MCF-7 sEVs, respectively (Fig. 7A). We also determined the effect of MCF-7-derived sEVs on cell migration. TAMR-MCF-7 cell migration was also enhanced by treatment with 16 μg/mL MCF-7 sEVs, but their efficacy was lower than that of TAMR-MCF-7 sEVs (4.1-fold vs. 7.2-fold increase)(Fig. 7B). However, MCF-7 cell migration was not affected by MCF-7-derived or TAMR-MCF-7-derived sEVs (Fig. 7C).

We also compared the effects of T47D sEV and TAMR-MCF-7 sEV on cell migration intensity of both the cell types in transwell migration assays because migration and invasion of both the cell types are related with P2X7 receptor. sEV isolated from T47D cells also enhanced cell migration of TAMR-MCF-7 cells, but cell migration of T47D cells was only marginally affected by TAMR-MCF-7 sEV or T47D sEV (Fig. 7D). Hence, the type of cell sensing sEV, rather than the type of cell that produces sEV, appears to be important for sEV-mediated cell migration.

## Discussion

Here, we investigated the role of purinergic signaling in TAMR-MCF-7 cells. The mRNA and protein expression of P2X7 was selectively upregulated in TAM-MCF-7 cells, and ATP-dependent Ca²^+^ influx was potently suppressed by KN62 in TAMR-MCF-7 cells but not in MCF-7 cells. Interestingly, we confirmed that P2X7 activation in TAMR-MCF-7 cells did not activate the classically known inflammasome pathways (IL-1β and IL-18), showing that P2X7 may control a non-canonical pathway in cancer cells. We could not detect ASC expression in TAMR-MCF-7 cells. P2X7 receptor is a well-known receptor causing programmed cell death such as pyroptosis via continuous stimulation of inflammasome^12,30–32^. Because P2X7 expressed in TAMR-MCF-7 cells is intensively activated relative to MCF-7 cells, the continuous stimulation of P2X7 in TAMR-MCF-7 cells may result in pyroptosis-mediated cell death as well as inflammasome activation^33^. So, we assume that ASC deficiency found in TAMR-MCF-7 cells is a kind of self-protection mechanism to prevent ATP-induced cell death by P2X7 receptor activation.

The activity of P2X7 in TAM-resistant breast cancer seemed to be confined to cell migration and metastasis rather than cell proliferation. In fact, several studies have reported that P2X7 receptor signaling induces growth-promoting activity and indicated that the P2X7 receptor plays a key role in cell proliferation^34,35^. In contrast, it has also been reported that the pro-apoptotic effect ensues from exogenous stimulation of P2X7 in several cell types^10,36^. Therefore, the function of the P2X7 receptor in cell proliferation is still controversial and has been actively debated. The role of the P2X7 receptor in proliferation may depend on cell type or extracellular ATP concentration^31^.

We then investigated the mechanism by which P2X7 affects the metastasis or migration of TAM-resistant breast cancer. KN62 treatment did not change the expression patterns of the EMT markers that play important roles in the metastasis of cancer cells. The only significant change was a decrease in Zeb-1 expression. Although Zeb-1 acts as a functional E-cadherin repressor^37^, Zeb-1 downregulation by the P2X7 antagonist did not coincide with recovery of E-cadherin in TAMR-MCF-7 cells. Cell migration and tumor metastasis are associated with the enhanced expression of metastasis-related genes, including MMPs, a family of proteinases^38^. In particular, the invasiveness of breast cancer is closely related with overexpression of MMP-2 and MMP-9^39^. Although the protein expression of MMP-2 and MMP-9 was highly upregulated in TAMR-MCF-7 cells, exposure of TAMR-MCF-7 cells to ATP or KN62 slightly affected the levels of both MMP types. Hence, the anti-migratory effect of the P2X7 antagonist may not result from inhibition of the EMT process or downregulation of MMPs.

Bi-directional interaction between the cell and its microenvironment is very important for normal tissue homeostasis and cancer cell growth^40^. Pre-metastatic niche is a concept that describes the cancerous microenvironment in which the growth of metastatic cancer cells migrated from a primary cancer can be promoted^41^. Vascular endothelial growth factor, cancer-derived sEVs, and components of sEV are known to be stimulating factors for pre-metastatic niche formation^42^. In particular, it has recently been noted that sEVs are involved in cellular or intercellular signaling^43^, and play a key role in the invasion and metastasis of breast cancer cells^40^. However, the molecular mechanisms through which sEVs promote metastasis remain incompletely understood. Recent studies have reported a possible link between the release of sEVs and activation of purinergic receptors. For example, activation of purinergic receptors with ATP can evoke the release of extracellular vesicles in the RAW264.7 mouse macrophage cell line^44^. Moreover, another study found that the P2X7 receptor is relevant to the release of sEVs in murine macrophages with brief stimulation by ATP^45^. Because intracellular calcium is required for the secretion of sEVs^27^, we hypothesized that P2X7 activity is critical for sEV secretion in TAM-resistant breast cancer. In fact, KN62 treatment significantly reduced the basal secretion of sEVs in TAMR-MCF-7 cells. Moreover, the expression of CD63, a membrane factor controlling the release of sEVs, was also decreased by KN62. It is noteworthy that P2X7 activity directly modulates the release of sEVs from cancer cells to promote migration and metastasis in TAM-resistant breast cancer.

In addition, Rab5 overexpression has been shown to inhibit both early endosomal trafficking and exosomal release of CD63^46^. Hence, in comparison to MCF-7 cells, overexpression of CD63 in TAMR-MCF-7 cells may be due to the decreased expression of Rab5. Although expression levels of CD63 and Rab5 are different in MCF-7 and TAMR-MCF-7 cells, the number of sEVs secreted from both the cell types was not distinct. In fact, diverse regulators including phosphoinositide phosphates and other Rab family members (Rab7, Rab11, Rab27, Rab35) are included in the biogenesis of exosomes^47^. For example, Rab7 depletion was known to impair release of exosome^47^. During the acquisition of TAM resistance in MCF-7 cells, the expression pattern of proteins required for the release of EV seems to be changed.

Even though the expression level of P2X7 in MDA-MB-231 cells was not enhanced relative to MCF-7 cells, the migration ability and CD63 expression was diminished in MDA-MB-231 cells by P2X7 antagonists (KN62 and oATP). The expression level of P2X7 seems not to be directly linked to the activity of P2X7 receptor. Despite of marginal expression of P2X7 in MCF-7 cells, ATP-driven calcium increase was not reversed by P2X7 antagonist in the cell type, indicating P2X7 is not active. It would be possible that ion channel activity of P2X7 in MDA-MB-231 cells is more sensitive than that in MCF-7 cells.

We further confirmed that the addition of sEVs isolated from either MCF-7 cells or TAMR-MCF-7 cells stimulated the migration of TAMR-MCF-7 cells. This result corresponds with previous studies showing that sEVs play a critical role in various cancer metastases^41^. However, sEVs from neither cell type caused cell migration in MCF-7 cells, implying that the existence of an sEV-sensing system is important for the migration of cancer cells. Compared to the understanding of sEV biogenesis and secretion, the uptake system of sEVs is still not well understood^42^. Although several studies have suggested that sEVs are taken up through clathrin-mediated endocytosis^48,49^, further research is needed to confirm this mechanism in TAM-resistant breast cancer. Our results raise the possibility that breast cancer cells evolve to express a recognition system of sEVs by acquiring TAM resistance.

Overall, we propose a novel role of the P2X7 receptor expressed in TAM-resistant breast cancer and describe a mechanism of metastasis through the secretion of sEVs. We demonstrated for the first time that the P2X7 receptor is selectively overexpressed in TAM-resistant breast cancer cells and actively involved in cancer cell migration and tumor metastasis *in vitro* and *in vivo*. More importantly, sEV secretion via P2X7 activation enhances the migration of TAMR-MCF-7 cells. It is noteworthy that ATP concentrations in the intercellular space near tumor tissues may reach 100 μM, but little is detectable in normal body tissues^50^. When breast cancer acquires TAM resistance, the highly expressed P2X7 receptor may be activated by ATP abundantly present in the cancer microenvironment to contribute to cancer metastasis. In turn, our data suggest that targeting the P2X7 receptor in TAM-resistant breast cancer cells may provide a gateway for metastasis prevention in TAM-resistant cancer patients in the clinic. Despite this value, there is a limitation to use only one clone as TAM-resistant breast cancer cell line (TAMR-MCF-7 cells), so further studies using various TAM-resistant cell lines and clinical samples will be necessary.

## Methods

### Cell culture and establishment of TAMR-MCF-7 cell

MCF-7 and MDA-MB-231 cells were cultured at 37 °C in 5% CO_2_/95% air in Dulbecco’s modified Eagle’s medium (DMEM) containing 10% fetal bovine serum (FBS) and antibiotics. T47D cells were cultured in RPMI medium containing 10% FBS. TAMR-MCF-7 cells were established as previously reported^51^.

### Antibodies and reagents

Antibodies against Zeb-1, horseradish peroxidase-conjugated donkey anti-rabbit and anti-mouse IgGs were purchased from Cell Signaling Technology (Beverly, MA). Antibodies recognizing E-cadherin and N-Cadherin were obtained from BD Bioscience (San Jose, CA). Antibodies against NLRP3 and ASC were purchased from Adipogen (Liestal, Switzerland). Antibodies targeting CD63, Rab5, vimentin, P2X7 and pro-Caspase-1 were supplied from Santa Cruz Biotech (Santa Cruz, Ca). Anti-snail antibody was purchased from Abcam (Cambridge, UK). Anti-actin antibody was obtained from Sigma (St. Louis, MO). Alexa Fluor 488 donkey anti-mouse IgG, Alexa Fluor 568 goat anti-rabbit IgG and Alexa Fluor 546 Phalloidin antibodies were purchased from Life Technologies (Gaithersburg, CA). sEV-depleted fetal bovine serum (FBS) and control FBS were purchased from Systembioscience (Mountain View, CA).

### Western blot analysis

Total cell lysates were prepared as previously reported^52^. After centrifugation of total cell lysates (13,000 *g* for 15 min), the supernatant were applied for immunoblottings. Enhanced chemiluminescence (ECL) system reagent (EMD Milipore, Billerica, MA) was used for band detection.

### Intracellular calcium determination

The intracellular calcium contents were measured using a FlexStation scanning fluorimeter with an integrated fluid transfer workstation (Molecular Devices, Sunnyvale, CA). 5 × 10^4^ cells were seeded in 96-well plates and cultured overnight. Then, the final volume was adjusted to 200 μL using the assay buffer of FLIPR Ca²^+^ assay kit (Molecular Devices), followed by incubation at 37 °C for 1 h. Using the FlexStation, the intracellular calcium concentration was measured after setting the excitation, emission, and cut-off at 485, 525 and 515 nm at 2.5-second intervals for 3 min.

### Total RNA isolation and reverse-transcriptase-polymerase chain reaction (RT-PCR) analysis

Total RNA was extracted from MCF-7 and TAMR-MCF-7 cells using TRIzol reagent (Life Technologies, Grand Island, NY) according to the manufacturer’s protocol. 1 μg total RNA was reverse-transcribed using Maxime RT-PreMix Kit (Intron biotechnology, Gyeonggi-do, Korea) to synthesize cDNA, and the obtained cDNA was amplified by PCR using a Maxime PCR PreMix Kit (Intron biotechnology, Gyeonggi-do, Korea) and electrophoresed on 2% agarose gel. Detailed primer sequences used in the experiments are provided as Supplementary Information (Supplementary Table S1). Quantitative polymerase chain reaction was carried out using SYBR green (biorad, San Francisco, CA) incorporation with gene-specific primers. The forward primer for P2X7 was: 5′- TATGAGACGAACAAAGTCACTCG-3′ and the reverse one was: 5′- GCAAAGCAAACGTAGGAAAAGAT-3′. The primers for 18 S were: forward: 5′- CCATCCAATCGGTAGTAGCG-3′; reverse: 5′- GTAACCCGTTGAACCCCATT-3′. Relative gene expression was calculated by ΔΔCt analysis relative to 18 S.

### Real-time monitoring of cell proliferation

5 × 10^4^ cells were seeded in 96 well-plate and the phase percentage of cells were scanned every 4 h by using the IncuCyte ZOOM^TM^ Live Cell Anaylsis System (Essen Bioscience, Ann Arbor, MI).

### Immunocytochemistry

Coverglass sterilized with 70% ethanol was placed on a 24-well plate and 1 × 10^5^ cells were seeded and the attached cells were incubated with vehicle or compound for 24 h. After washing twice with PBS, the cells were fixed with 4% paraformaldehyde for 20 min at room temperature and incubated with 0.1% Triton X-100 for 15 min. After washing three times with PBS, the fixed and permeable cells were incubated with 10% horse serum for 1 h, and the cells were exposed to the primary antibody at 4 °C overnight and subsequently fluorophore-conjugated secondary antibody. Then, the coverglass was mounted on a slide glass and fixed with mounting gel, and the fluorecence images were obtained using iRiS ™ Digital Cell Imaging System (Logos Biosystems, Gyeonggi-do, Korea).

### Transwell migration and wound healing assays

Cell migration assay was quantified by both trans-well migration and wound healing assays. For transwell migration assay, MCF-7 and TAMR-MCF-7 cells were seeded in the upper chamber of the trans-well plate and the lower chamber was filled with 10% FBS-containing media. The cells were incubated at 37 °C for 18 h and then fixed with 4% formalin and methanol, and subsequently stained with hematoxylin and eosin. With 40× magnification, migrated cells to the lower filter side were analyzed. Also some parts of transwell migration assay were performed using Incucyte Zoom^TM^ Live Cell Analysis System (Essen bioscience) with using Incucyte clearview chemotaxis plate. For wound-healing assay, cells were seeded at 4 × 10^4^ cells/well in 96-well ImageLock plate (Essen bioscience) and the cells were incubated until 100% confluency. 96-well WoundMaker (Essen bioscience) was used to make consistent scratch. After adding 100 μL culture media to 96-well plate, wound scratch was photographed every 4 h using Incucyte Zoom and the change pattern was analyzed.

### P2X7 siRNA and transfection

To improve the gene silence efficacy, two different P2X7 receptor siRNA (Bioneer, Daejeon, Korea) were combined: (1) sense:5′-CUCUUGAGGAGCGCCGAAA-3′; antisense:5′-UUUCGGCGCUCCUCAAGAG-3′ (2) sense: 5′-CAGUGAAUGAGUACUACUA‐3′; antisense: 5′-UAGUAGUACUCAUUCACUG‐3′. The scrambled siRNA was used as the control. 200 pmol P2X7 receptor siRNA or scrambled siRNA, was transfected using Lipofectamine 2000 (Life Technologies, Carlsbad, Ca) according to the manufacturer’s protocol.

### Spleen-liver metastasis and pathological assessment

All experiments and methods were performed in accordance with relevant guidelines and regulations. All animal procedures were approved by the Institutional Animal Care and Use Committee of Seoul National University (Approval #: SNU-160412-1-1). Five weeks old BALB/c athymic nude mice (Raon Bio Inc., Seoul, Korea) were inoculated with 1 × 10^6^ TAMR-MCF-7 cells in spleen. 10 mm subcoastal incision was made in order to expose the spleen. The half of spleen was tied with nylon thread to prevent leakage of injecting cells. The suspended cells were injected into the spleen and the spleen was restored back into abdominal cavity. The incisions were closed with nylon thread. After 14 days, KN-62 was intraperitoneally injected three times a week for additional 6 weeks. Liver tissues were fixed in formalin for histological analysis. Incidence of liver metastasis and metastasis area were analyzed by certified pathologist.

### Enzyme-linked immunosorbent assays (ELISA)

Human IL-1β and IL-18 ELISA kits were purchased from Enzo life sciences (Farmingdale, NY). Culture media were 80 times concentrated by using Amicon Ultra-4 Centrifugal Filter Units (Merck Milipore, Darmstadt, Germany) and ELISA was carried out according to the provided manuals.

### sEV isolation and particle size determination

MCF-7 and TAMR-MCF-7 cells were cultured on 150 mm^2^ dishes and washed twice with PBS. After incubation of cells with FBS-free culture media for 24 h, culture media were collected, and the media were serially centrifuged to remove dead cells and debris. (300 g for 10 min, 2500 g for 20 min, 10000 g for 30 min). 200 nm filtration was performed to remove vesicles and contaminants larger than the sEV size. The resulting supernatant was subjected to ultracentrifugation [120000 g for 90 min, Optima XE-100 with 32Ti rotor, Beckman Coulter (Miami, FL)]. The supernatants were discarded for removal of contaminating proteins, and then the tube was filled with PBS and subjected to ultracentrifugation (120000 g for 90 min). The resident sEV pellet was solubilized in sterile PBS. Intensity, volume, and numerical distribution of the isolated sEVs were analyzed by ELSZ-1000 (Photal Otsuka Electronics, Osaka, Japan).

### Nanoparticle tracking analysis

Nanosight LM10, version 3.0 (Malvern Instruments, Malvern, UK) was used to determine the number of cell-secreted sEVs. The detection conditions are as follows. Sample dilution: HPLC grade water; Camera level: 7; Detection threshold: 5; Detection time: 30 s.

### Statistical analysis

Densitometry scanning was performed using Multi gauge software (Fujifilm, Tokyo, Japan). Student’s t-test or one-way analysis of variance (ANOVA) were used to assess statistical significance.

## Supplementary information

Supplementary information
