# Supplementary material for: Involvement of the P2X7 receptor in the migration and metastasis of tamoxifen-resistant breast cancer: effects on small extracellular vesicles production
Source: Sci Rep. 2019 Aug 12;9:11587. doi: 10.1038/s41598-019-47734-z (PMC6690963; doi:10.1038/s41598-019-47734-z)
Supplement: Supplementary file 1 — Supplementary information [file 41598_2019_47734_MOESM1_ESM.pdf]

Involvement of the P2X7 receptor in the migration and metastasis of tamoxifen-resistant breast cancer: effects on small extracellular vesicles production

Miso Park[a,§], Jieun Kim[a,§], Nguyen TT Phuong[a], Jung Gyu Park[a], Jin-Hee Park[b], Yong-Chul Kim[b], Moon Chang Baek[c], Sung Chul Lim[d], Keon Wook Kang[a,*]

Supplemental Information

Figure S1.

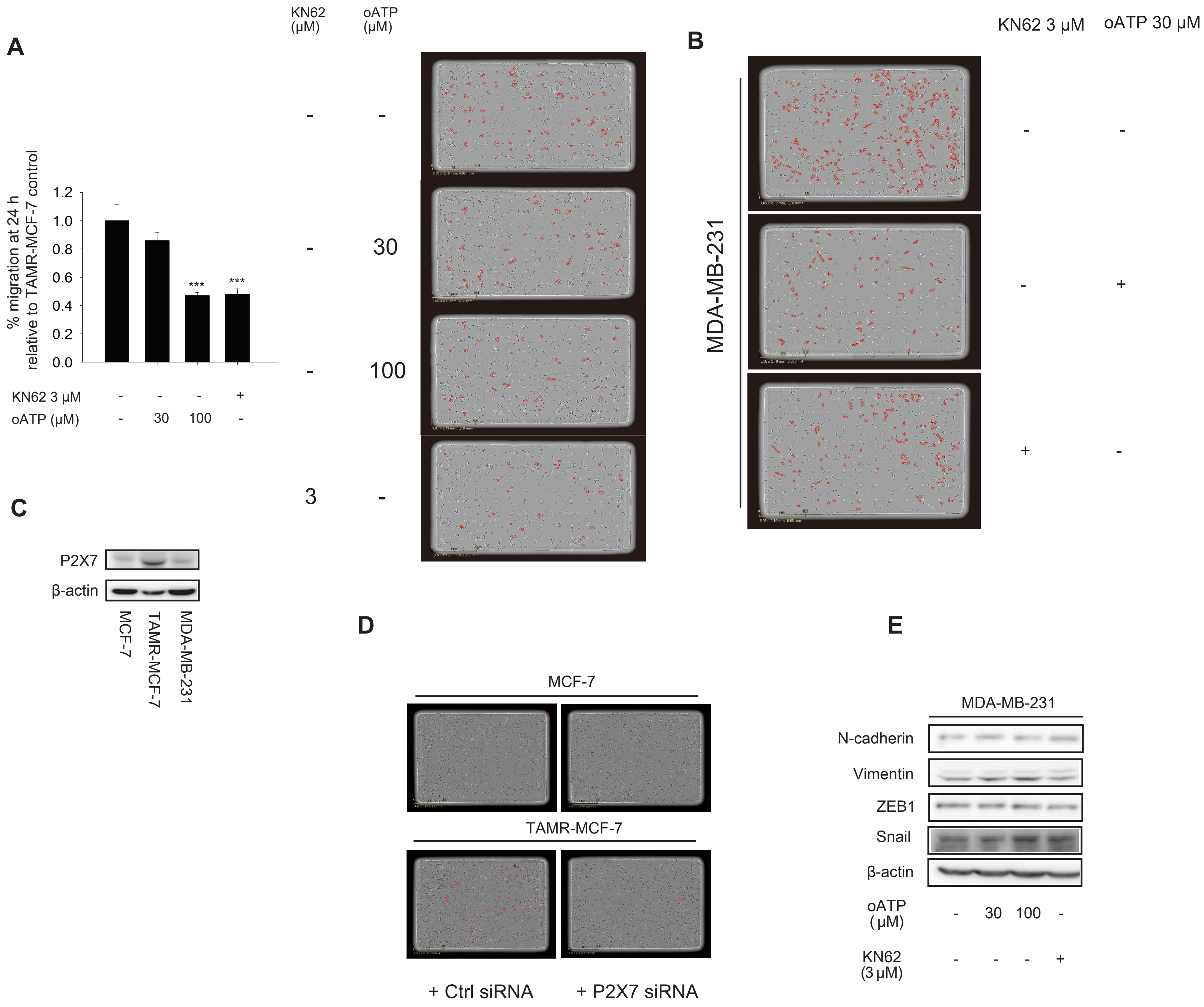
A
KN62 (μM)
oATP (μM)
% migration at 24 h relative to TAMR-MCF-7 control
1.2
1.0
0.8
0.6
0.4
0.2
0.0
***
***
KN62 3 μM - - - +
oATP (μM) - 30 100 -
- -
- 30
- 100
3 -
B
KN62 3 μM
oATP 30 μM
MDA-MB-231
- -
- +
+ -
C
P2X7
β-actin
MCF-7
TAMR-MCF-7
MDA-MB-231
D
MCF-7
TAMR-MCF-7
+ Ctrl siRNA
+ P2X7 siRNA
E
MDA-MB-231
N-cadherin
Vimentin
ZEB1
Snail
β-actin
oATP (μM) - 30 100 -
KN62 (3 μM) - - - +

Figure S1. (A) Effect of oATP (30 and 100 μM) and KN62 (3 μM) on the basal cell migration of TAMR-MCF-7 cells (n=3). (B) Effect of oATP (30 μM) and KN62 (3 μM) on the basal cell migration of MDA-MB-231 cells (n=4). Migrated cells were marked in red by image analysis. (C) Protein expression of P2X7 in MCF-7, TAMR-MCF-7 and MDA-MB-231 cells. (D) Effects of P2X7 silencing on cell migration of MCF-7 and TAMR-MCF-7 cells (n=4). Migrated cells were marked in pink by image analysis. Images were taken by Incucyte Zoom. (E) Expression of EMT markers (N-cadherin, Vimentin, Zeb-1, and Snail) in MDA-MB-231 cells after treatment with oATP or KN62 for 48 h. All data represent the mean ± SE (***p<0.005, significant as compared to TAMR-MCF-7 control cells).

Figure S2.

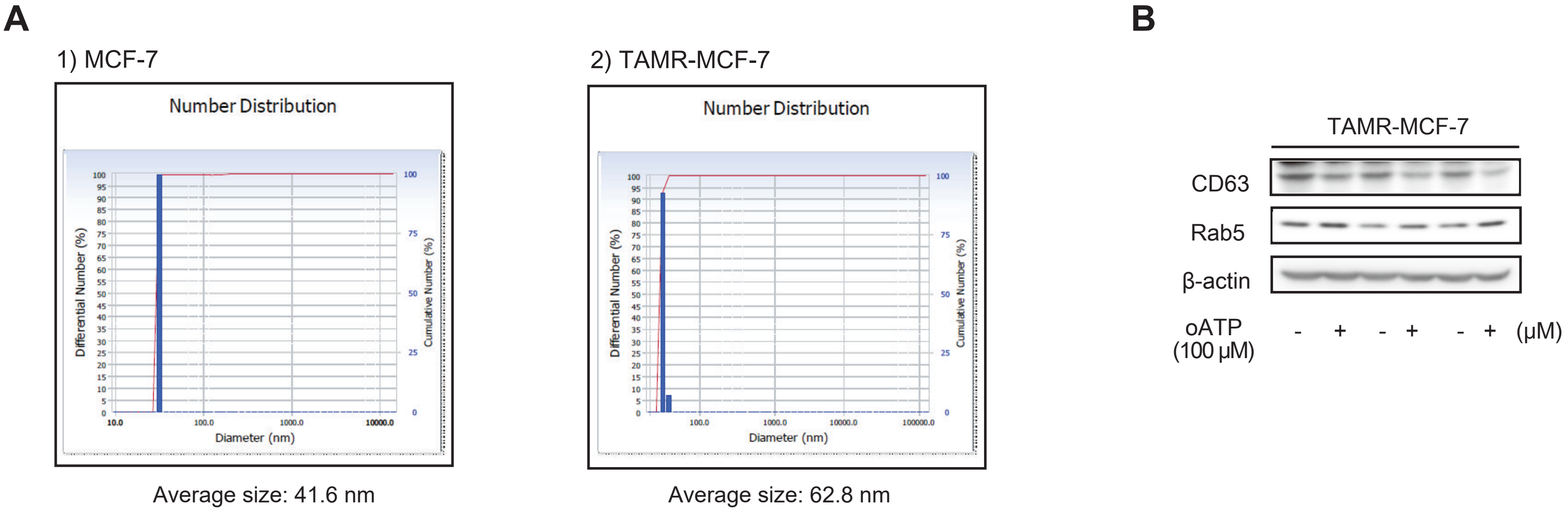
A
1) MCF-7
Number Distribution
Differential Number (%)
Cumulative Number (%)
Diameter (nm)
Average size: 41.6 nm
2) TAMR-MCF-7
Number Distribution
Differential Number (%)
Cumulative Number (%)
Diameter (nm)
Average size: 62.8 nm
B
TAMR-MCF-7
CD63
Rab5
β-actin
oATP (100 μM)
- + - + - + (μM)

Figure S2. (A) Measurement of secreted exosome number distribution in MCF-7 and TAMR-MCF-7 cells by direct light scattering. Average diameter was calculated from each peak. (B) Effects of oATP on the protein expression of CD63 and Rab5 in TAMR-MCF-7 cells.

Figure S3.

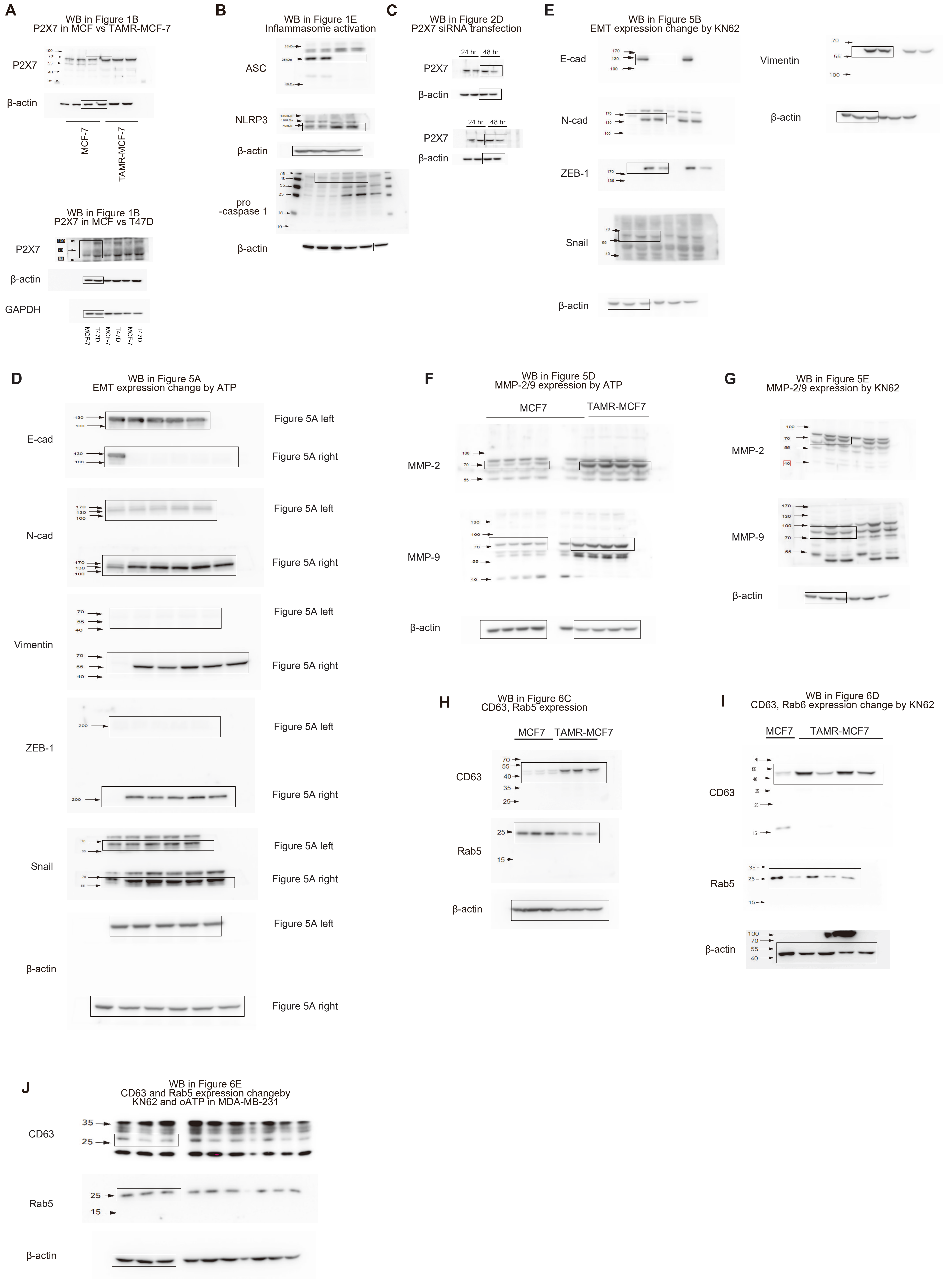
A
WB in Figure 1B
P2X7 in MCF vs TAMR-MCF-7
P2X7
β-actin
MCF-7
TAMR-MCF-7
WB in Figure 1B
P2X7 in MCF vs T47D
P2X7
β-actin
GAPDH
MCF-7
T47D
MCF-7
T47D
MCF-7
T47D
B
WB in Figure 1E
Inflammasome activation
ASC
NLRP3
β-actin
pro
-caspase 1
β-actin
C
WB in Figure 2D
P2X7 siRNA transfection
24 hr
48 hr
P2X7
β-actin
24 hr
48 hr
P2X7
β-actin
E
WB in Figure 5B
EMT expression change by KN62
E-cad
N-cad
ZEB-1
Snail
β-actin
Vimentin
β-actin
D
WB in Figure 5A
EMT expression change by ATP
E-cad
Figure 5A left
Figure 5A right
N-cad
Figure 5A left
Figure 5A right
Vimentin
Figure 5A left
Figure 5A right
ZEB-1
Figure 5A left
Figure 5A right
Snail
Figure 5A left
Figure 5A right
β-actin
Figure 5A left
Figure 5A right
F
WB in Figure 5D
MMP-2/9 expression by ATP
MCF7
TAMR-MCF7
MMP-2
MMP-9
β-actin
G
WB in Figure 5E
MMP-2/9 expression by KN62
MMP-2
MMP-9
β-actin
H
WB in Figure 6C
CD63, Rab5 expression
MCF7
TAMR-MCF7
CD63
Rab5
β-actin
I
WB in Figure 6D
CD63, Rab6 expression change by KN62
MCF7
TAMR-MCF7
CD63
Rab5
β-actin
J
WB in Figure 6E
CD63 and Rab5 expression changeby
KN62 and oATP in MDA-MB-231
CD63
Rab5
β-actin

Figure S3. Full-length pictures of the blots presented in Figures1,2,5,6.

Supplementary Table S1. Sequences of primer used

Gene	Forward Primer 5’ – 3’	Reverse Primer 5’ – 3’
hP2X1	TTTCATCGTGACCCCGAAGCAG	TCAAAGCGAATCCCAAACACC
hP2X2	ACCTGCCCCGAGAGCATAAG	AATGACCCCGATGACACCACCC
hP2X3	CACCTCGGTCTTTGTCATCATCAC	TGTTGAACTTGCCAGCATTCC
hP2X4	ACAGCAACGGAGTCTCAACAGG	CCTTCCCAAACACAATGATGTCG
hP2X5	AACCTGATTGTGACCCCCAACC	TCGCAGAAGAAAGCACCCTTGC
hP2X6	GGTGACCAACTTCCTTGTGACG	CCCAGTGAACTCTGATGCCTACAG
hP2X7	TGCGATGGACTTCACAGATTTG	TGCCCTTCACTCTTCGGAAAC
hP2Y1	CCGGCTGTCTACATCTTGGT	GGCAGAGTCAGCACGTACAA
hP2Y2	CCACCTGCCTTCTCACTAGC	TGGGAAATCTCAAGGACTGG
hP2Y4	TGCCTGGTCACTCTTGTTTG	GTACTCGGCAGTCAGCTTCC
hP2Y6	CGACCACATGAGCTCCTACA	GAGCTTCTGGGTCCTGTGAG
hP2Y11	AGGGCAAAGTGATGTTCCAC	CCCTCCAGGCTCTTCTTTCT
hP2Y12	AACTGGGAACAGGACCACTG	ACATGAATGCCCAGATGACA
hP2Y13	TCGTGGCTGTCTTCTTTGTG	TTTCTTGGCTTGATGCTGTG
hP2Y14	TTAAAAGGCCTCTGCCTTCA	AGAGCTGGGCACGTAAAAGA
hGAPDH	CATCAGAAGTATGACAACAGCC	AGTCCTTCCACGATACCAAAG
18s rRNA	TCCAAGGGTCCGCTGCAGTC	CGTTCACCTTGATGAGCCCA
